# P-830. A Simple Score, A Big Difference: Antimicrobial Stewardship using the Bladder Health Score in Resource-Limited Regions

**DOI:** 10.1093/ofid/ofaf695.1038

**Published:** 2026-01-11

**Authors:** Suhail Hassan Jalal, Roshni Murali, Eiphan Kezia, Suja Koshi, Ravi Kirtana

**Affiliations:** The Tamil Nadu Dr. M.G.R. Medical University, Chennai, Tamil Nadu, India; The Tamil Nadu Dr. M.G.R. Medical University, Chennai, Tamil Nadu, India; Madras Medical Mission, Chennai, Tamil Nadu, India; The Madras Medical Mission Hospital, Chennai, Tamil Nadu, India; The Madras Medical Mission Hospital, Chennai, Tamil Nadu, India

## Abstract

**Background:**

Inappropriate prescribing of antibiotics in people with nonspecific urinary symptoms is a challenge in low-resource rural areas, where diagnostic limitations worsen antimicrobial resistance. The Bladder Health Score (BHS), a validated clinical tool differentiates true urinary tract infections (UTIs) from asymptomatic bacteriuria, providing a stewardship opportunity. This study evaluated a clinical pharmacist-led intervention to reduce inappropriate antibiotic use in elderly patients of Rural South Indian villages.

**Methods:**

A prospective study was conducted from July 2024 to March 2025 in two demographically similar villages. One served as the intervention village, where clinical pharmacists implemented the BHS to guide antibiotic decision-making; the other served as the control village, where patients received standard care. Participants included were elderly residents (≥60 years) presenting with urinary symptoms, delirium or altered mental status. In the intervention village, pharmacists implemented BHS assessments (score ≥3 indicating UTI likelihood) and provided real-time antibiotic recommendations to providers. Primary outcome was inappropriateness of antibiotic prescriptions. Secondary outcomes were days of therapy with antibiotics (DOT), empirical-culture concordance and readmissions at 30 days

**Results:**

Of 462 enrolled patients (intervention: 234; control: 228), the intervention village demonstrated:

- 58% reduction in inappropriate antibiotic prescriptions (OR 0.42, 95% CI 0.29–0.61; *p*< 0.001).
- Lower mean antibiotic DOT (4.2 vs. 7.1 days; *p*=0.003) with comparable clinical cure rates (93.0% vs. 91.7%).
- 89% positive predictive value of BHS ≥3 for culture-confirmed UTI.
- 87.3% provider acceptance rate of pharmacist recommendations.
- No significant difference in 30-day readmissions (4.7% vs. 5.2%; *p*=0.78).

**Conclusion:**

This pharmacist-delivered BHS intervention strongly decreased rural elderly populations' inappropriate antibiotic use, proving to be feasible and clinically safe. The high predictive value of BHS and strong provider acceptance underscore its utility in low-resource settings. By aligning with WHO AMR priorities, this model offers a cost-effective stewardship strategy for regions with diagnostic limitations.

**Disclosures:**

All Authors: No reported disclosures

